# Decision algorithm for when to use the ICD-11 3-part model for healthcare harms

**DOI:** 10.1186/s12911-022-01887-6

**Published:** 2022-06-07

**Authors:** Cathy A. Eastwood, Shahreen Khair, Danielle A. Southern

**Affiliations:** 1 Centre for Health Informatics, Cumming School of Medicine, University of Calgary, 3280 Hospital Drive NW, TRW 5E06, Calgary, AB T2N 4Z6 Canada; 2 Department of Community Health Sciences, Cumming School of Medicine, University of Calgary, Calgary, Canada

**Keywords:** Patient safety, ICD-11, ICD, Harms, Decision-tree, 3-part model

## Abstract

Accurate data collection of healthcare-related adverse events provides a foundation for quality and health system improvement. The International Classification of Diseases for Mortality and Morbidity Statistics, 11th revision (ICD-11 MMS) includes new codes to identify harm or injury and the events or actions leading to the adverse events. However, it is difficult to choose the correct codes without in-depth understanding of which event may be classified as an injury or harm. A 3-part model will be available in the ICD-11 MMS to cluster the codes for the harm or injury that occurred, the causal factors, and the mode (mechanism) involved. While field testing coding of adverse events, our team developed a decision tree (algorithm), which guides when to use the 3-part model. The decision tree now resides in the ICD-11 Reference Guide. This paper is part of a special ICD-11 paper series and outlines the steps used in the decision-tree (algorithm) and provides examples to help understand the process.

While it may take coders some time to gain experience to use the 3-part model and decision-tree, the ICD-11 Reference Guide and this paper can be helpful resources to help clarify the process.

## Background

Healthcare quality is of utmost importance for patient safety. Without accurate data collection for surveillance of healthcare-related adverse events, quality gaps cannot be identified, nor system improvements made. It has been said that without measurement, improvement is not possible [1]. Medical incident reporting is one method for recording adverse events in real-time. Coding events post-hospital discharge can provide another measurement vehicle for surveillance and case identification for further scrutiny when warranted. Measurement involves careful discernment of what constitutes hospital-related harm or not. The current International Classification of Diseases 10^th^ revision (ICD-10) has limited detail, some overlapping codes, and some conceptual gaps that can limit coding of healthcare-related harms [2]. The 11th revision of ICD for Mortality and Morbidity Statistics (ICD-11) was released to the public in 2018 by the World Health Organization [3] and offered new features conducive to capturing healthcare-related harms.

**Fig. 1:**
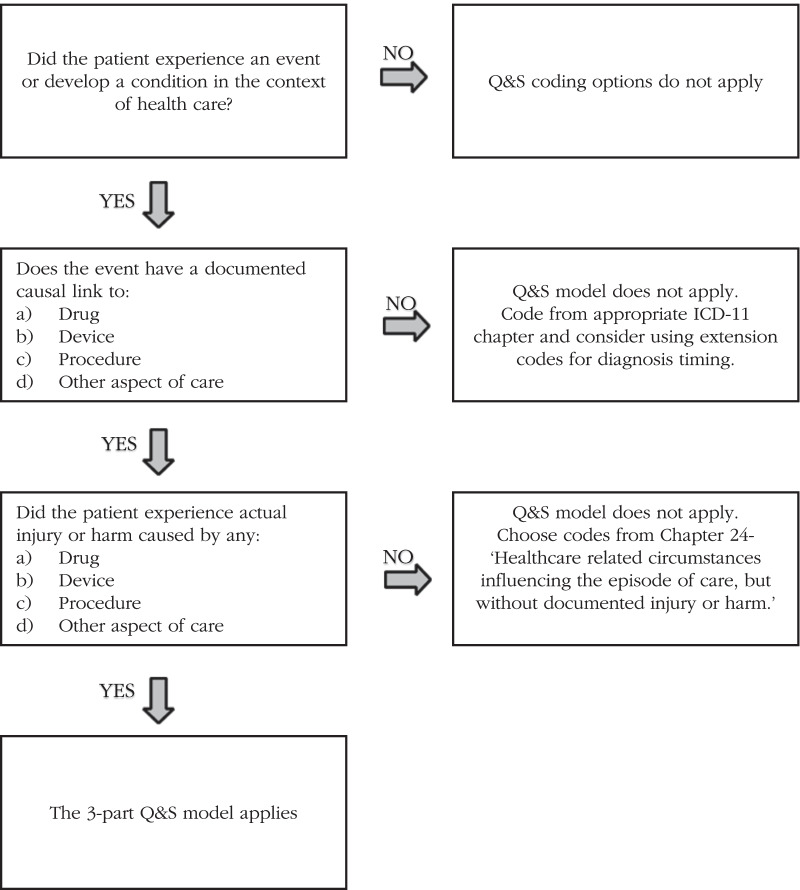
Decision-tree for when to use the 3-part Quality & Safety (Q&S) model. Source: Adapted from ICD-11 Reference Guide Sect. 2.25.17.4 [4]

During the World Health Organization’s (WHO) ICD-11 revision process, a 3-part model emerged to capture the harm or injury that occurred, the causal factors, and the mode (mechanism) that was involved [4]. The 3-part model is a framework to record detailed descriptions of healthcare-related quality and safety events. From experience of coding during a large ICD-11 field trial [5, 6], in conjunction with a WHO consultant, careful attention has gone into developing reference materials to guide the decision process for coding healthcare-related harms. This paper is part of a special series that illustrates features within the ICD-11 coding system and their corresponding reference materials in the ICD-11 Reference Guide [4]. This is the 4th paper in a sequence of papers discussing the 3-part model. The other 3 papers are: (1) The three-part model for coding causes and mechanisms of healthcare-related adverse events [7], (2) Coding of adverse health care events that do not harm patients (in draft) [8], and (3) Interpreting and coding causal relationships for quality and safety using ICD-11 (under review) [9]. This paper describes resources available to coders, informing them on how to record adverse events in a variety of situations (Fig. 1).

## Main text

### Methods and results: coding using the 3-part model

Coding a healthcare-related adverse event is not always easy to understand. Sometimes, adverse events occur without a specific causal link to healthcare and minimal context about how it occurred (mechanism). Thus, it is vital to distinguish when to code the adverse event as healthcare-related harm. The ICD-11 3-part model helps to identify an adverse event with an established causal link to healthcare. The model captures: (i) a cause of the injury or harm related to a healthcare activity, (ii) a mode or mechanism of injury or harm which is related to the cause and (iii) the harmful consequences to the patient, resulting from the adverse event. Healthcare-related harm or injury causes are categorized into four types: 1. substances (drugs, medicaments and biological substances); 2. procedures (surgical and other); 3. devices (surgical and other devices, implants or grafts); and 4. other healthcare-related causes (e.g., problems associated with the physical transfer of patient, non-provision of necessary procedure, delayed diagnosis, fall in health care, etc.) as discussed in an earlier paper in the series. The mode of harm further describes how the harm occurred by which mechanism if it was documented. Modes typically are action oriented. Some examples of modes are ‘perforation,’ ‘malfunction,’ ‘mismatched blood,’ or ‘dropped patient.’

The new code definitions in ICD-11 and reference materials include more clarity regarding language around causation as well as codes available for near-miss situations when no injury takes place [4]. When injury or harmful consequences of an event to the patient occur, in the online browser for ICD-11[10], codes can be selected from Chapters 1 to 22 of ICD-11, which cover a broad array of ailments. Codes can be found in Chapter 23 for capturing External Causes of Healthcare-related Harm or Injury, with subsections (code identifiers PK and PL) for causes and modes of the harm.

Before coding is possible, one needs to determine if healthcare-related harm or injury has occurred and when to use the 3-part model. A large real-world field trial was conducted to test ICD-11 codes along with ICD-10 and chart review data collection from 3011 full hospital records [6]. One of the focuses of the study was on the quality and safety code chapters and training coders using the new 3-part model for healthcare-related harms. During this study, the coding team mapped the thought processes that guided their decisions for when to use the 3-part model for coding healthcare-related harms. This became a decision-tree which is in part 2.25.5- ‘Overview of code-set in ICD–11 for quality and patient safety’ [4] (World Health Organization, 2019)of the online ICD-11 reference guide [4].

### How and when to use the 3-part model

The new content in ICD-11 for adverse events has been developed using these two theoretical foundations—the International Classification for Patient Safety (ICPS) [11] and the AHRQ Common Formats [12]. The 3-part model has been described in detail in the companion article in this series [7] and in a previously published article on adverse event detection [13]. As previously mentioned in the series article that describes the 3-part model [7], the model applies when the following 3 conditions are met and are documented:

- The patient (medical inpatient or outpatient) experienced an event or developed a condition in the context of healthcare.
- The event or condition is documented and has a causal link to a drug, device, procedure, or any other aspect of care.
- The patient experienced actual injury or harm caused by a drug, device, procedure, or any other aspect of care.

If all three conditions are met, in addition to the condition code (code for the harm or injury), codes for cause and mode would be chosen from Chapter 23, External Causes of Morbidity and mortality (Causes of healthcare-related harm or injury). If an event does occur, but no explicit causal link to a device, drug, procedure or other aspects of care is documented, then a code would be chosen that describes the event or situation experienced by the patient (e.g., fall) and no further codes are required. If no injury or harm occurs after a healthcare-related event, then a Chapter 24 code may apply (Health care related circumstances influencing the episode of care without injury or harm). Examples of each of these situations, when the 3-part model does not apply, are provided below.

### Examples of when to use the 3-part model

Below are two examples and descriptions of when and how to use the 3-part model.

#### Example 1: Adverse event with harm, cause, mode

**Table:** 

Patient admitted for a laparoscopic cholecystectomy for gallbladder calculus and chronic cholecystitis. During removal of the gallbladder from the fossa, a minor laceration of the liver occurred, which was sutured
**NB91.11/PK80.32/PL11.0** **NB91.11** *Laceration of liver, minor* **PK80.32** *Gastrointestinal, abdominal, or abdominal wall procedure associated with injury or harm, endoscopic approach* **PL11.0** *Cut, puncture or tear as mode of injury or harm*

This example shows how the 3-part model is to be used. When using the Decision Tree, there is evidence that ‘Yes,’ the patient developed a condition in the context of healthcare: a laceration of the liver. A causal link to a procedure is also present with the wording ‘during removal of the gallbladder.’ Thirdly, the mode is described in the documentation as a laceration which is coded as a cut, puncture, or tear. Once the laceration of the liver is searched in the Coding Tool [15], the cause (procedure) and mode (tear) can be post-coordinated using the search function for the box labelled ‘associated with.’ All 3 steps of the Decision Tree are answered with ‘Yes’; hence the 3-part model applies to be coded.

A code string is produced with the stem code first (harm), then the cause code, then the mode, and any other description such as timing or location. The forward slash is used to cluster and string the codes together.

#### Example 2: Adverse event with harm, cause, mode

**Table:** 

Female patient was found with bleeding evident from her urethra. Upon inspection, a laceration of the urethra was seen caused by dislodgement of the urinary catheter
**NB92.31/PK93.10/PL12.4&XY69** **NB92.31 Laceration of urethra** **PK93.10** Gastroenterology or urology devices associated with adverse incidents, **urinary catheter** **PL12.4 Dislodgement,** misconnection, or de-attachment, as mode of injury or harm **XY69** Developed after admission

Similarly, this clinical example has evidence of healthcare-related harm. The decision-tree helps confirm the decision and guides thinking and searching for all 3 parts of the code cluster. The harm is the laceration, the cause is the device, and the dislodgment of the device was the mode by which the injury occurred.

### The coding tool and 3-part model decisions

**Fig. 2:**
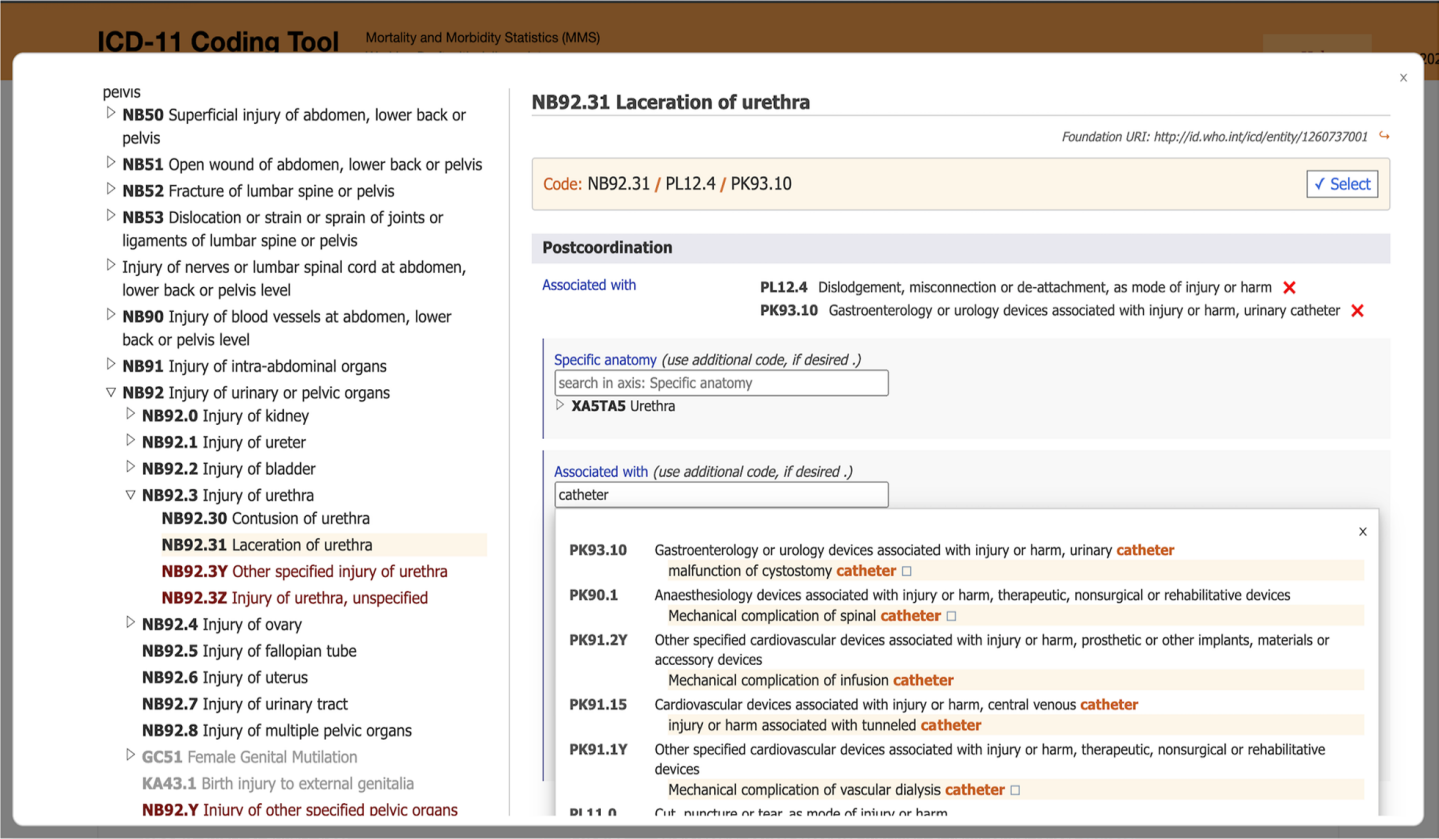
Screenshot of the ICD-11 Coding Tool showing post coordination for ‘Laceration of urethra.’ Source: ICD-11 Coding Tool [15]

The ICD-11 Coding Tool offers useful quick search features for events and conditions by suggestion to look for what might be ‘associated with’ the condition and points the coder towards potential causes and modes. The following screenshot indicates the steps for coding using the Coding Tool for Example 2 above (Fig. 2). By first typing in *Laceration of urethra*, then ‘associated with’ *catheter* and then again with *dislodgement,* all 3 parts of the code cluster can be built from within the Coding Tool.

### Examples of when *not to* use the 3-part model

When a clear causal link cannot be established for a health condition, i.e., when detailed documentation is unavailable or ambiguous, the decision-tree can help make the determination that the 3-part model would not apply. An example of this would be ‘atrial fibrillation after surgery.’ The usage of descriptive words like ‘after,’ ‘occurring on day XX,’ or ‘following,’ do not indicate that the factors played a causal role. Such conditions would be coded using a medical condition from any chapter from ICD-11 and include an extension code for timing, diagnosis arising during a hospital stay, etc. [10]. The example below would be coded in the following way, and the 3-part model would not apply.

#### Example 3: Adverse event but no clear causal link

**Table:** 

After a knee replacement, Mr. Jones developed atrial fibrillation on day 3 after surgery. He became symptomatic and required treatment
**BC81.3& XT5R& XY69& XY7V** **BC81.3** Atrial fibrillation **XT5R** Acute **XY69** Developed after admission **XY7V** Postoperative

In this example, the decision-tree first step is answered with ‘Yes’ because the condition that occurred while in hospital was Atrial Fibrillation. However, the second question, ‘Does the event or condition have a documented causal link to a drug, device, procedure, or other aspect of care?’ is answered with ‘No.’ We know the Atrial Fibrillation began after surgery, but it is not clear that the surgery *caused* the condition. Without a clear causal link to some aspect of healthcare service, the 3-part model does not apply, and the condition is just described with applicable codes for timing and related circumstances (postoperative).

#### Example 4: Adverse event but no harm (near miss)

**Table:** 

After leaving the bathroom, Mrs. Smith became weak and fell to the floor. Found sitting, alert and oriented. No complaints of pain, no bruises, lacerations, or other evidence of injury
QA8E Fall in health care without documented injury or harm

Lastly, mishaps can occur in healthcare settings without resulting in harm or injury (see examples 4 and 5). Harm or injury is coded when the patient requires intervention (e.g., sutures) or treatment (e.g., antibiotics), an amended treatment course (e.g., 6 weeks of home IV antibiotics), the length of hospital stay was lengthened related to the harm, or a specialist was consulted. If none of these sequelae occur, a ‘near miss’ may have occurred. As such, codes from Chapter 24: Factors influencing health status or contact with health services, section QA that covers “Circumstances associated with a surgical or other medical procedure influencing the episode of care, without injury or harm.” (8).

#### Example 5. Adverse drug event but no harm (near miss)

**Table:** 

Upon leaving the hospital, Mr. Brown was given a prescription for a new statin drug that seemed to have a different name from the one he was taking. He took both medication for 2 weeks. One was the generic name; one was the trade name. The family physician realized that Mr. Brown was taking double the recommended dose and stopped one drug. Bloodwork was checked and there was no rise in CK levels. Mr. Brown denied myopathy or other evidence of injury when the nurse called to check on him 24 h later
QA70 Overdose of a substance without injury or harm

## Conclusions

Using the decision-tree of the 3-part model helps identify and code harm or patient safety event more accurately. The 3-part model also helps add information about the circumstances which may have contributed to the injury. The decision tree helps gather the proper information and simplifies the decision points for coders. It is vital to distinguish between which medical event can be classified as harm and which cannot be classified as harm. Information for cause and mode can be grouped as healthcare-related harm and used for quality improvement purposes [4]. Future harms may be avoided if the actions which led to the injury or harm are documented and studied.

The concepts in the decision tree can be globally applied to any healthcare setting where harm or injury may occur. The decision-tree highlights the decision points and would be universally applicable to inpatient, outpatient, rural, or urban settings. With the availability of multiple language translations being incorporated, the usability and international applicability of the decision-tree and the 3-part model is increasing.

The decision-tree for using the 3-part model requires further evaluation for refinement. Further testing and evaluation of the 3-part model are required as reference material detail and coding tools, as we describe, have been significantly updated. The algorithmic logic may not capture all scenarios or ‘what if?’ situations. However, the simple three questions were sufficient to guide the coders in a large field trial as the coders gained experience coding hospital-related harms. At times, causal link language is not always clearly written. Then, the coders cannot assume the 3-part model applies and should simply code the condition (harm) and timing of the event (before or during hospital stay). No matter which measurement method is employed, medical terminologies (e.g., SNOMED) or ontologies for disease classification (e.g., ICD-11), or medical incident reporting systems, all methods depend on the quality of the clinical notes, on interventions for improved quality of care, and a three-part framework is useful [14].

Coders may require some experience and training to become adept at identifying when to use the 3-part model and how to use the decision-tree. Both the decision-tree in the ICD-11 Reference Guide [4] and the Coding Tool [15] guide decision making. When documentation is clear, the post coordination in the Coding Tool can lead the coder to the codes for the cause and mode, for building more descriptive code clusters than possible with ICD-10.

The ICD-11 Reference Guide [4] offers more detailed examples (2.25.4 to 2.25.5.), and the Coding Tool [15] guides post-coordination. The ICD-11 Reference Guide provides an explanatory text which helps coders to understand which codes to choose. As demonstrated in the ICD-11 Field Trial, when coders are given a wide variety of real-world examples and adequate practice time, proficiency can be gained. While learning to code healthcare harms, these coders appreciated discussions with their peers to correctly apply the decision tree concepts and locate the correct codes. [6]

Great advances have been made in the ICD-11 MMS for more precise coding of healthcare-related harms. A simple decision-tree has been provided in the ICD-11 Reference Guide to support how to effectively apply the 3-part model (harm, cause, mode) and determine when it does not apply. This decision-tree was developed and refined collaboratively by coders and quality/safety experts as a tool for enhancing the coding of such events.

## Data Availability

The cohorts discussed here cannot be shared publicly, but agreements to have them analyzed can be made in cooperation with Alberta Health Services and The Centre for Health Informatics at the University of Calgary (https://cumming.ucalgary.ca/centres/centre-health-informatics/data-and-analytic-services/data-resources/requesting-ahs-data-resources).

## Abbreviations

ICD: International Classification of Diseases
ICD-10: International Classification of Diseases, 10th revision
ICD-11: International Classification of Diseases, 11th revision
